# Institution-Specific Machine Learning Models for Prehospital Assessment to Predict Hospital Admission: Prediction Model Development Study

**DOI:** 10.2196/20324

**Published:** 2020-10-27

**Authors:** Toru Shirakawa, Tomohiro Sonoo, Kentaro Ogura, Ryo Fujimori, Konan Hara, Tadahiro Goto, Hideki Hashimoto, Yuji Takahashi, Hiromu Naraba, Kensuke Nakamura

**Affiliations:** 1 Department of Public Health Graduate School of Medicine Osaka University Suita Japan; 2 TXP Medical Co, Ltd Chuo-ku Japan; 3 Department of Emergency Medicine Hitachi General Hospital Hitachi Japan; 4 Faculty of Medicine The University of Tokyo Bunkyo-ku Japan; 5 Department of Public Health The University of Tokyo Bunkyo-ku Japan; 6 Department of Clinical Epidemiology and Health Economics School of Public Health The University of Tokyo Bunkyo-ku Japan; 7 Department of Emergency Medicine The University of Tokyo Bunkyo-ku Japan

**Keywords:** prehospital, prediction, hospital admission, emergency medicine, machine learning, data science

## Abstract

**Background:**

Although multiple prediction models have been developed to predict hospital admission to emergency departments (EDs) to address overcrowding and patient safety, only a few studies have examined prediction models for prehospital use. Development of institution-specific prediction models is feasible in this age of data science, provided that predictor-related information is readily collectable.

**Objective:**

We aimed to develop a hospital admission prediction model based on patient information that is commonly available during ambulance transport before hospitalization.

**Methods:**

Patients transported by ambulance to our ED from April 2018 through March 2019 were enrolled. Candidate predictors were age, sex, chief complaint, vital signs, and patient medical history, all of which were recorded by emergency medical teams during ambulance transport. Patients were divided into two cohorts for derivation (3601/5145, 70.0%) and validation (1544/5145, 30.0%). For statistical models, logistic regression, logistic lasso, random forest, and gradient boosting machine were used. Prediction models were developed in the derivation cohort. Model performance was assessed by area under the receiver operating characteristic curve (AUROC) and association measures in the validation cohort.

**Results:**

Of 5145 patients transported by ambulance, including deaths in the ED and hospital transfers, 2699 (52.5%) required hospital admission. Prediction performance was higher with the addition of predictive factors, attaining the best performance with an AUROC of 0.818 (95% CI 0.792-0.839) with a machine learning model and predictive factors of age, sex, chief complaint, and vital signs. Sensitivity and specificity of this model were 0.744 (95% CI 0.716-0.773) and 0.745 (95% CI 0.709-0.776), respectively.

**Conclusions:**

For patients transferred to EDs, we developed a well-performing hospital admission prediction model based on routinely collected prehospital information including chief complaints.

## Introduction

For patients being transported to an emergency department (ED), predicting hospital admission is important for providing high-quality care. Choosing the appropriate destination hospital with available beds can enhance efficient resource utilization in the context of integrated community health care [1]. Furthermore, accurate risk stratification during transportation can be expected to curb the risk of ED overcrowding and reduce ambulance turnaround times when implemented at hospitals [2].

Although multiple prediction models have been developed to predict hospital admission for ED use [3-11] to address overcrowding and patient safety [12-15], few studies have examined prediction models for prehospital use. Previously reported prehospital prediction models have been limited to patients with a specific disease or to models predicting critically ill conditions or mortality [16-23]. Several studies in the United States and United Kingdom have demonstrated the predictive performance of ED disposition, including hospitalization for general patients transferred by ambulance [24-26]. Nevertheless, these studies were not based on statistical models but on subjective prediction by ambulance staff. Therefore, they have limited generalizability across emergency medical systems and countries. Another study, conducted in Sweden, assessed a prehospital prediction model of hospital admission [27]. However, its predictors included more than 1000 distinct question and answer combinations recorded in a clinical decision support system used at a dispatch center. Therefore, its scalability might not be readily achievable.

Given this context, we aimed to develop prehospital prediction models of hospital admission using machine learning techniques and conventional logistic regression, based on replicable measurements such as chief complaints, vital signs, and past medical histories, which can all be collected routinely in an ambulance in any country. Our goal was to develop an institution-specific model based on readily collectable data with sufficient predictive performance, not a universal model that has broad generalizability.

## Methods

### Study Design and Setting

This prognostic study used data obtained at a tertiary care hospital in Japan from April 2018 to March 2019. The hospital covers approximately 3 million local residents. Annually, the hospital has about 20,000-25,000 visits, including 5500-6500 ambulance visits. The study protocol was approved by the Ethics Committee of the hospital. They waived informed consent because of the characteristics of the retrospective study design.

### Study Participants

We enrolled patients who had been transported to our ED by ambulance. We excluded children aged 6 years or younger because of the difficulty in taking chief complaints and measuring vital signs such as blood pressure. Patients with cardiopulmonary arrest were not excluded from analyses, thereby facilitating comparison with earlier studies that included patients with cardiopulmonary arrest and examined the predictive performance of ambulance staff [24-26].

### Patient Information in the Prehospital Setting

Vital signs were measured at the scene when the patient was placed in the ambulance. After emergency medical service (EMS) staff members recorded patient information and conditions during transportation, they transmitted the information via telephone to ED staff members at the destination hospital. This information was input into an ED database through the Next Stage ER system (TXP Medical Co, Ltd), which structures information related to the chief complaint and past medical history with flexible input templates and a minor natural language processing algorithm [28]. The recorded chief complaint was translated automatically into 231 chief complaint categories based on the Japan Triage and Acuity Scale (JTAS) [29], which was developed based on the Canadian Triage and Acuity System [30]. Past medical histories were encoded corresponding to the International Statistical Classification of Diseases, 10th Revision (ICD-10) codes [31].

### Candidate Predictors

Candidate predictors were age, sex, chief complaints, prehospital vital signs, and past medical histories. Although chief complaints were grouped into 231 categories based on JTAS, 75 complaints were not observed (ie, none of the included patients presented with these complaints). Therefore, 156 complaints were used. Vital signs include the level of consciousness, systolic blood pressure, diastolic blood pressure, pulse rate, respiratory rate, body temperature, and oxygen saturation with oxygen administration during transportation. The level of consciousness was assessed according to the Japan Coma Scale, which can be summarized briefly into four categories of alert, possible eye opening but not lucid, possible eye opening upon stimulation, and no eye opening and coma [32]. Past medical histories were grouped using the first 3 characters (1 alphabet letter and 2 digits) of the ICD-10 code. The 156 chief complaints and 505 past medical histories observed in our study were encoded to dummy variables. In all, 832 predictors were identified as candidate predictors.

### Outcome Measures

The primary outcome was the composite of hospitalization, transfer to other care facilities, and death at the ED. These outcomes were recorded at the time patients left the ED. Sensitivity analysis was performed by excluding mortality from the hospitalization outcomes.

### Data Analysis

#### Model Development

To predict hospital admission, we developed four models using candidate predictors as explained above: (1) logistic regression, (2) logistic regression with lasso penalization (logistic lasso), (3) random forest [33], and (4) gradient boosting machine (GBM) [34]. For the GBM model, we used the extreme gradient boosting (XGBoost) implementation [35]. For each model, to evaluate the incremental benefit of adding each predictor, we further developed four models according to the predictors. Model 1 consists of age and sex only. Model 2 further includes 156 chief complaints. Model 3 further includes vital signs. Model 4 further includes 505 past medical histories. These modalities were designed according to the typical temporal order of information collection processing: call by a patient or bystander, arrival of an emergency medical team, and examination in the ambulance.

#### Feature Processing

To account for potential nonlinear relations between continuous features and the risk of hospital admission, we categorized the values of age and vital signs into deciles for logistic regression and logistic lasso. Since random forest and GBM can accommodate the nonlinear relations, we used continuous age and vital signs in those models.

#### Study Cohorts and Missing Values

We used 70.0% (3601/5145) of the available data for the derivation cohort. The remaining 30.0% (1544/5145) of data were used for the validation cohort. We divided patients into the two groups by random allocation. Hyperparameters for machine learning models were determined using a grid search with 5-fold cross-validation in the derivation cohort. Among the 5145 patients, frequencies (proportions) of missing values were 25 (0.5%) for sex, 552 (10.7%) for orientation, 593 (11.5%) for systolic blood pressure, 647 (12.6%) for diastolic blood pressure, 511 (9.9%) for pulse rate, 1152 (22.4%) for respiratory rate, 1040 (20.2%) for oxygen saturation, and 1086 (21.1%) for body temperature. The number of patients with at least one missing vital sign was 2174 (42.3%). To address the missing data, we used a missing indicator for logistic regression and lasso, assigned 0 for random forest, and left missing data in GBM, for which XGBoost can accommodate missing values.

#### Model Validation

In the validation cohort, we examined the prediction ability of the models by calculating the area under the receiver operating characteristic curve (AUROC) and the area under the precision-recall curve (AUPRC). Calibration of the models was depicted by plotting predicted probabilities and the observed admission rates according to deciles of the predicted probabilities. Sensitivity, specificity, positive predictive value, negative predictive value, and accuracy were estimated with predictors in the most accurate model at the threshold probability that maximizes the Youden indices [36].

For comparison with earlier studies of hospital admission prediction in the ED including walk-in patients and those transported by ambulance [3-11], we evaluated the prediction performance of the model described above including walk-in patients.

All analyses were conducted using Python 3.7 with scikit-learn [37], XGBoost [35], and tableone [38] packages. We used 200 bootstrap samples to calculate 95% confidence intervals for performance measures. Two-tailed *P* values of <.05 were inferred as statistically significant.

## Results

During the study period, 5530 patients were transported to our ED by ambulance. From these, we excluded 385 visits by patients aged 6 years or younger. In all, 5145 visits were included in the analyses. Among the 5145 visits with ambulance transport, 2507 visits (48.7%) led to hospital admission, 96 visits (1.9%) led to death in the ED, and 96 visits (1.9%) required hospital transfer. The number of patients who required hospital admission, died in the ED, or required hospital transfer was 1889 of 3601 patients (52.5%) in the derivation cohort and 810 of 1544 patients (52.5%) in the validation cohort. Compared to patients who were not admitted to the hospital, patients who were admitted to the hospital (including those who died or were transferred) had worse vital signs (eg, lower level of consciousness, lower blood pressure). Moreover, they were older, were likely to have altered mental status or fever, and were likely to have a history of circulatory and respiratory system symptoms (Table 1).

**Table 1:** Baseline characteristics of patients according to hospital admission status.

Characteristic				Admission		*P* value	
				No (n=2446)	Yes (n=2699)		
Age (years), mean (SD)				63.0 (23.7)	73.4 (16.2)		<.001
Male sex, n (%)				1308 (53.5)	1591 (58.9)		.25
**Selected chief complaint, n (%)^a^**							
	Altered mental status			220 (9.0)	373 (13.8)		<.001
	Dyspnea			176 (7.2)	403 (14.9)		.75
	Chest pain			188 (7.7)	200 (7.4)		.54
	Abdominal pain			169 (6.9)	174 (6.4)		<.001
	Fever			97 (4.0)	175 (6.5)		<.001
**Vital signs**							
	**Level of consciousness, n (%)**						
			Alert	1619 (66.2)	1338 (49.6)		<.001
			Possible eye opening, not lucid	427 (17.5)	642 (23.8)		<.001
			Possible eye opening upon stimulation	85 (3.5)	228 (8.4)		<.001
			No eye opening and coma	36 (1.5)	218 (8.1)		<.001
	Systolic blood pressure (mm Hg), mean (SD)			148.0 (33.2)	140.7 (43.8)		<.001
	Diastolic blood pressure (mm Hg), mean (SD)			83.5 (21.2)	80.8 (27.5)		<.001
	Pulse rate (bpm), mean (SD)			88.7 (21.3)	92.7 (24.7)		<.001
	Respiratory rate (bpm), mean (SD)			21.5 (5.3)	22.8 (6.2)		<.001
	Body temperature (°C), mean (SD)			36.8 (7.6)	36.9 (2.7)		.76
	Oxygen saturation (%), mean (SD)			97.0 (3.1)	93.8 (7.7)		<.001
	Oxygen administration during transportation, n (%)			287 (11.7)	947 (35.1)		<.001
**Selected past medical history, n (%)^a^**							
	R09		Other symptoms and signs involving the circulatory and respiratory systems	624 (25.5)	805 (29.8)		<.001
	E11		Type 2 diabetes mellitus	419 (17.1)	582 (21.6)		<.001
	I63		Cerebral infarction	202 (8.3)	303 (11.2)		.001
	E78		Disorders of lipoprotein metabolism and other lipidemias	151 (6.2)	212 (7.9)		.02
	I51		Complications and ill-defined descriptions of heart disease	159 (6.5)	180 (6.7)		.85

^a^The five most frequent chief complaints and past medical history items are shown.

**Figure 1:**
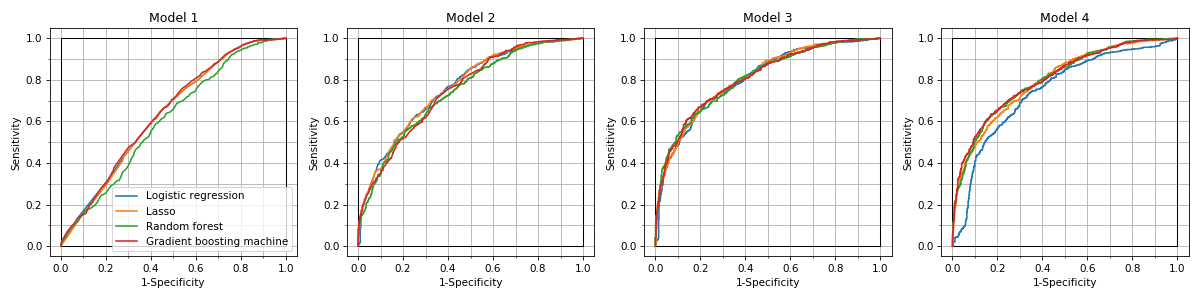
ROC curves of hospital admission prediction models. ROC curves for the three machine learning models are similar to those of logistic regression in models 1, 2, and 3, but superior to those of logistic regression in model 4. ROC: receiver operating characteristic.

**Figure 2:**
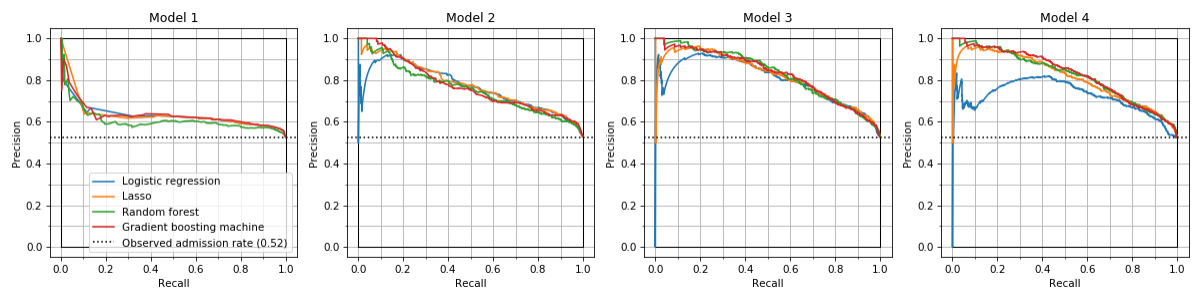
Precision-recall curves of hospital admission prediction models. Precision-recall curves of the three machine learning models are similar. Logistic regression model showed inferior performance for patients with higher predicted probabilities (left side on the horizontal axis) in models 2, 3, and 4.

**Figure 3:**
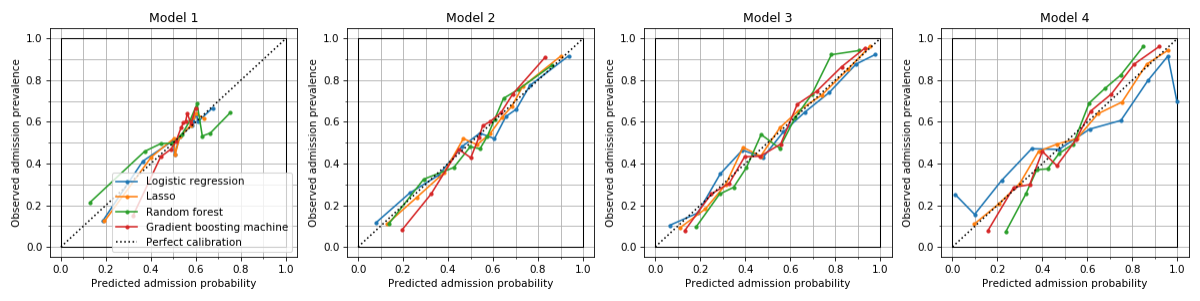
Calibration curves of hospital admission prediction models. Lasso and gradient boosting machine showed good calibration in all models. Logistic regression was ill-calibrated for patients with the lowest and the highest deciles of predicted probability in model 4.

Overall, the GBM model achieved the highest AUROCs and AUPRCs in models 3 and 4 (Tables 2 and 3). The most accurate model was GBM in model 3, with AUROC of 0.818 (95% CI 0.792-0.839), AUPRC of 0.831 (95% CI 0.804-0.855), sensitivity of 0.744 (95% CI 0.716-0.773), and specificity of 0.745 (95% CI 0.709-0.776) (Tables 2-4). The highest AUROC of logistic regression was 0.805 (95% CI 0.782-0.827) in model 3. It was lower in model 4: 0.750 (95% CI 0.720-0.774) (Figure 1). In models 2-4, precision-recall curve analysis showed superior performance of machine learning models compared to that of logistic regression among patients with higher risk of hospital admission (Figure 2). The lasso and GBM showed good calibration in all models (Figure 3). Hyperparameters of machine learning models are shown in Table S1 in Multimedia Appendix 1. The exclusion of mortality at the ED showed slightly lower predictive performance, with AUROC of 0.803 (95% CI 0.775-0.823) for GBM in model 3 (Tables S2-S4 in Multimedia Appendix 1).

**Table 2:** Areas under the receiver operating characteristic curve and 95% confidence intervals of hospital admission prediction models according to machine learning methods and prediction models.

Model type	Model 1^a^	Model 2^b^	Model 3^c^	Model 4^d^
Logistic regression	0.631 (0.602-0.657)	0.750 (0.723-0.774)	0.805 (0.782-0.827)	0.750 (0.720-0.774)
Lasso	0.631 (0.602-0.657)	0.755 (0.730-0.779)	0.817 (0.793-0.839)	0.811 (0.787-0.832)
Random forest	0.594 (0.567-0.619)	0.735 (0.710-0.763)	0.813 (0.786-0.834)	0.814 (0.786-0.833)
Gradient boosting machine	0.624 (0.598-0.652)	0.758 (0.734-0.783)	0.818 (0.792-0.839)	0.815 (0.788-0.833)

^a^Model 1: Age and sex.

^b^Model 2: Age, sex, and chief complaint.

^c^Model 3: Age, sex, chief complaint, and vital signs.

^d^Model 4: Age, sex, chief complaint, vital signs, and past medical history.

**Table 3:** Areas under the precision-recall curve and 95% confidence intervals of hospital admission prediction models according to machine learning models and predictor modalities.

Model type	Model 1^a^	Model 2^b^	Model 3^c^	Model 4^d^
Logistic regression	0.614 (0.578-0.653)	0.729 (0.700-0.766)	0.794 (0.764-0.827)	0.709 (0.667-0.744)
Lasso	0.614 (0.578-0.654)	0.766 (0.737-0.795)	0.829 (0.805-0.853)	0.820 (0.793-0.845)
Random forest	0.580 (0.550-0.615)	0.734 (0.703-0.770)	0.828 (0.802-0.853)	0.828 (0.801-0.851)
Gradient boosting machine	0.609 (0.580-0.647)	0.766 (0.734-0.799)	0.831 (0.804-0.855)	0.828 (0.803-0.852)

^a^Model 1: Age and sex.

^b^Model 2: Age, sex, and chief complaint.

^c^Model 3: Age, sex, chief complaint, and vital signs.

^d^Model 4: Age, sex, chief complaint, vital signs, and past medical history.

**Table 4:** Measures of predictive performance and 95% confidence intervals for prediction model 3 at optimal thresholds^a^.

Model type	Sensitivity	Specificity	PPV^b^	NPV^c^	Accuracy
Logistic regression	0.760 (0.724-0.786)	0.731 (0.698-0.766)	0.752 (0.728-0.783)	0.737 (0.700-0.771)	0.746 (0.723-0.770)
Lasso	0.724 (0.684-0.751)	0.774 (0.742-0.800)	0.776 (0.748-0.803)	0.722 (0.683-0.752)	0.749 (0.723-0.767)
Random forest	0.720 (0.687-0.749)	0.777 (0.742-0.804)	0.776 (0.745-0.807)	0.718 (0.685-0.748)	0.745 (0.720-0.768)
Gradient boosting machine	0.736 (0.696-0.768)	0.743 (0.712-0.772)	0.757 (0.726-0.785)	0.721 (0.680-0.756)	0.739 (0.713-0.765)

^a^Predictors were age, sex, chief complaint, and vital signs.

^b^PPV: positive predictive value.

^c^NPV: negative predictive value.

A GBM model with data of both walk-in and ambulance visits to our ED during the study period (n=16,857) demonstrated higher performance than that for patients transported by ambulance, with AUROC of 0.873 (95% CI 0.860-0.883), sensitivity of 0.830 (95% CI 0.807-0.850), and specificity of 0.743 (95% CI 0.712-0.772) in the validation set.

## Discussion

### Principal Findings

To our knowledge, this report is the first of a study developing and validating prediction models for hospital admission based on common prehospital information for patients transported to EDs by ambulance. Information used for this study was collected in prehospital settings within a routine clinical practice. Therefore, the method of the prediction model development is readily applicable to other facilities that support clinical decision making by EMSs.

Our results are comparable to those presented in earlier reports describing the performance of subjective prediction by ambulance staff for patients they transported. A prospective study in the United Kingdom revealed a response rate of 99.7% (396/397). Analyses of 396 cases demonstrated sensitivity of 0.717 (95% CI 0.65-0.78) and specificity of 0.770 (95% CI 0.71-0.81) [24]. Another prospective study conducted in the United States found a response rate of 24.6% (101/411) from the cases analyzed [25]. Sensitivity of prediction by EMS staff members was 0.733 (95% CI 0.658-0.798), and the specificity was 0.850 (95% CI 0.798-0.891). Another study in the United States examined 932 transports to a hospital and reported the performance of EMS staff prediction of hospitalization as 0.62 (95% CI 0.54-0.68) for sensitivity and 0.89 (95% CI 0.86-0.91) for specificity [26]. However, prediction by EMS staff in this study was done at the time they left the ED. The results might be affected by incorporation bias because of observation or direct discussion with physicians and nurses in the ED. Therefore, the true performance might be lower. These studies are based on the impressions of paramedics. Therefore, their performance in other emergency medical systems remains unknown. However, our method relies on common prehospital measurements, which present the benefit of applicability to other standard emergency medical systems.

The AUROC achieved using the proposed model was lower than those reported from earlier studies for patients after arrival at the ED, reporting values of 0.80-0.87 [3-11]. However, these earlier models included both walk-in and ambulance patients. Because our prediction model was restricted to patients transferred by ambulance, the target population might be more severely affected by health issues than walk-in patients, making it difficult to discriminate patients who need inpatient care and patients who do not. Indeed, prediction performance including both walk-in and ambulance visits to our ED demonstrated comparable performance to that of an AUROC of 0.873.

The logistic regression model demonstrated comparable performance to that obtained with other machine learning models, with <0.02 difference in AUROCs in models 1-3 and lower performance in model 4. Two recent reports have described similar predictive performance in logistic regression and machine learning models for predicting hospital admission after ED visits [39,40]. However, the ratios of the number of variables to the number of patients were smaller in those studies than in this study: previous studies reported 972 variables to 560,486 patients [39], and 111 variables to 1,721,294 patients [40], whereas this study reported 832 variables to 5145 patients. The lower predictive performance of logistic regression can be attributed to overfitting. By selecting important predictors by lasso or other methods, a logistic regression model might be built with comparable performance to those of other machine learning models, as suggested by our result obtained for lasso, which virtually reduces the number of variables in logistic regression.

### Limitations

First, hospital admission might reflect not only the medical conditions, but also the social context. Performance can be improved by adding socioeconomic factors such as activities of daily living, education, income, type of insurance, family structure, and marital status, or neurological characteristics such as cognitive function and depressive symptoms, especially for elderly people [41-43]. Second, because the models were developed from data from a single institution, the external validity of our model is uncertain. For generalization of our results to other hospitals, assessments similar to ours are expected to be necessary. However, data used for this study can be collected automatically in daily routine practice. Therefore, development of a hospital-specific prediction model is feasible. For small hospitals with ED volume that is too small to generate a model, privacy-preserving federated learning [44,45] might provide a solution. Third, information on past medical history might be affected by information bias because it is collected in a critical situation. Nonsignificant incremental benefits of adding past medical history information in this study can be partially attributable to this bias. Accurate data collection of past medical history, for example, linkage to personal health care records in an integrated community health care network, might improve the model’s predictive performance. Fourth, we did not have detailed information related to the accurate time of measurement of vital signs. Taking the best or worst value of vital signs may increase the predictive ability of our proposed models.

### Conclusions

We developed a model of hospital admission prediction for patients transferred by ambulance using common prehospital information that performed well. The methodology used for this study can be extended to multicenter settings to facilitate efficient medical resource use in communities.

## Appendix

Multimedia Appendix 1 Supplementary tables.

## Abbreviations

AUPRC: area under the precision-recall curve
AUROC: area under the receiver operating characteristic curve
ED: emergency department
EMS: emergency medical service
GBM: gradient boosting machine
ICD-10: International Statistical Classification of Diseases, 10th Revision
JTAS: Japan Triage and Acuity Scale
XGBoost: extreme gradient boosting
