# Supplementary material for: Institution-Specific Machine Learning Models for Prehospital Assessment to Predict Hospital Admission: Prediction Model Development Study
Source: JMIR Med Inform. 2020 Oct 27;8(10):e20324. doi: 10.2196/20324 (PMC7655472; doi:10.2196/20324)
Supplement: Multimedia Appendix 1 [file medinform_v8i10e20324_app1.docx]

| Table S1. Hyperparameters for machine learning models determined by five-fold cross validation | | |
| --- | --- | --- |
| Model and Hyperparameter | | Value |
| Lasso | |  |
|  | Regularization coefficient | 1.0 |
| Random forest | |  |
|  | Number of trees | 200 |
|  | Max depth | 25 |
| Gradient boosting machine | |  |
|  | Number of estimators | 150 |
|  | Max depth | 4 |
|  | Subsample rate | 0.85 |
|  | Learning rate | 0.1 |
|  | Regularization coefficient of *l*1 penalty in post processing | 0.01 |

| Table S2. Areas under the receiver–operator characteristics curve and 95% confidence intervals of hospital admission prediction models according to machine learning methods and prediction models after the exclusion of mortality at the emergency department | | | | |
| --- | --- | --- | --- | --- |
|  | Model 1 | Model 2 | Model 3 | Model 4 |
| Logistic regression | 0.619 (0.589–0.644) | 0.741 (0.715–0.766) | 0.793 (0.767–0.816) | 0.740 (0.710–0.763) |
| Lasso | 0.619 (0.591–0.645) | 0.747 (0.723–0.772) | 0.805 (0.781–0.825) | 0.797 (0.774–0.819) |
| Random forest | 0.578 (0.552–0.604) | 0.724 (0.701–0.750) | 0.800 (0.776–0.820) | 0.800 (0.772–0.819) |
| Gradient boosting machine | 0.609 (0.582–0.636) | 0.746 (0.722–0.774) | 0.803 (0.775–0.823) | 0.804 (0.777–0.823) |
| Model 1: Age and sex. Model 2: Age, sex, and chief complaints. Model 3: Age, sex, chief complaints, and vital signs. Model 4: Age, sex, chief complaints, vital signs, and past medical histories. | | | | |

| Table S3. Areas under the precision–recall characteristics curve and 95% confidence intervals of hospital admission prediction models according to machine learning models and predictor modalities after the exclusion of mortality at the emergency department | | | | |
| --- | --- | --- | --- | --- |
|  | Model 1 | Model 2 | Model 3 | Model 4 |
| Logistic regression | 0.588 (0.548–0.628) | 0.704 (0.672–0.743) | 0.768 (0.737–0.804) | 0.689 (0.646–0.727) |
| Lasso | 0.587 (0.547–0.628) | 0.737 (0.700–0.771) | 0.803 (0.775–0.830) | 0.792 (0.762–0.817) |
| Random forest | 0.545 (0.516–0.581) | 0.701 (0.663–0.736) | 0.805 (0.779–0.831) | 0.799 (0.771–0.827) |
| Gradient boosting machine | 0.579 (0.547–0.617) | 0.731 (0.694–0.768) | 0.799 (0.772–0.830) | 0.800 (0.771–0.830) |
| Model 1: Age and sex. Model 2: Age, sex, and chief complaint. Model 3: Age, sex, chief complaint, and vital signs. Model 4: Age, sex, chief complaint, vital signs, and past medical history. | | | | |

| Table S4. Measures of predictive performance and 95% confidence intervals for prediction model 3 at the optimal thresholds after the exclusion of mortality at the emergency department | | | | | |
| --- | --- | --- | --- | --- | --- |
|  | Sensitivity | Specificity | PPV | NPV | Accuracy |
| Logistic regression | 0.710 (0.673–0.738) | 0.762 (0.731–0.790) | 0.751 (0.720–0.780) | 0.722 (0.683–0.752) | 0.736 (0.708–0.760) |
| Lasso | 0.702 (0.666–0.729) | 0.785 (0.756–0.812) | 0.767 (0.736–0.797) | 0.722 (0.687–0.749) | 0.744 (0.718–0.762) |
| Random forest | 0.697 (0.666–0.727) | 0.771 (0.741–0.797) | 0.755 (0.724–0.780) | 0.716 (0.684–0.749) | 0.734 (0.710–0.755) |
| Gradient boosting machine | 0.744 (0.712–0.772) | 0.716 (0.685–0.745) | 0.727 (0.697–0.755) | 0.734 (0.702–0.766) | 0.731 (0.705–0.752) |
| Predictors were age, sex, chief complaints, and vital signs. | | | | | |
| Abbreviations: PPV, positive predictive value; NPV, negative predictive value. | | | | | |
